# Improved quality of life in head and neck cancer patients treated with modern arc radiotherapy techniques – A prospective longitudinal analysis

**DOI:** 10.3389/fonc.2024.1424034

**Published:** 2024-09-23

**Authors:** Eva Yu-Hsuan Chuang, Pei-Yu Hou, Pei-Wei Shueng, Wu-Chia Lo, Ping-Yi Lin, Shih-Chiang Lin, Po-Hsuan Wu, Jing-Gu Jiang, Chen-Shuan Chung, Chen-Xiong Hsu, Deng-Yu Kuo, Yueh-Feng Lu, Li-Jen Liao, Chen-Hsi Hsieh

**Affiliations:** ^1^ Division of Radiation Oncology, Department of Radiology, Far Eastern Memorial Hospital, New Taipei, Taiwan; ^2^ Department of Computer Science and Engineering, Yuan Ze University, Taoyuan, Taiwan; ^3^ School of Medicine, College of Medicine, National Yang Ming Chiao Tung University, Taipei, Taiwan; ^4^ Head and Neck Cancer Surveillance and Research Group, Far Eastern Memorial Hospital, New Taipei, Taiwan; ^5^ Department of Otolaryngology Head and Neck Surgery, Far Eastern Memorial Hospital, New Taipei, Taiwan; ^6^ Graduate Institute of Medicine, Yuan Ze University, Taoyuan, Taiwan; ^7^ Department of Dentistry, Far Eastern Memorial Hospital, New Taipei, Taiwan; ^8^ General Education Center, Lunghwa University of Science and Technology, Taoyuan, Taiwan; ^9^ Graduate Institute of Clinical Dentistry, School of Dentistry, National Taiwan University, Taipei, Taiwan; ^10^ Division of Hematology and Oncology, Department of Internal Medicine, Far Eastern Memorial Hospital, New Taipei, Taiwan; ^11^ Department of Electrical Engineering, College of Electrical and Communication Engineering, Yuan Ze University, Taoyuan, Taiwan; ^12^ Institute of Toxicology, College of Medicine, National Taiwan University, Taipei, Taiwan; ^13^ Division of Gastroenterology and Hepatology, Department of Internal Medicine, Far Eastern Memorial Hospital, New Taipei, Taiwan; ^14^ College of Medicine, Fu Jen Catholic University, New Taipei, Taiwan; ^15^ Institute of Traditional Medicine, College of Medicine, National Yang Ming Chiao Tung University, Taipei, Taiwan

**Keywords:** head and neck cancer, quality of life, radiation therapy (radiotherapy), VMAT (volumetric modulated arc therapy), tomotherapy, arc radiation therapy

## Abstract

**Purpose:**

The present longitudinal study aimed to evaluate the potential impact of modern radiotherapy (RT) techniques on quality of life (QOL) in patients with head and neck (HNC) cancer.

**Materials and methods:**

In this single-center prospective study, participants were asked to complete QOL questionnaires that included the EORTC QLQ-C30, QLQ-H&N 35 and utility score by time trade-off (TTO) at three time points (2 weeks, 3 months and 6 months) after completion of RT. All patients were treated by modern RT techniques [volumetric modulated arc therapy (VMAT) or helical tomotherapy (HT)]. Patients who developed recurrence or died before the 6-month follow-up were excluded. Linear mixed models with random intercepts for participants and restricted maximum likelihood estimates were used to assess the effect of our study variables (age, sex, primary site, cancer stage, treatment, radiation dose and radiation method). Overall changes in QOL, utility scores and symptom burdens at different time points were tested using paired t tests.

**Results:**

A total of 45 patients were recruited from 2022 to 2023. Those who completed the surveys at 2 weeks with at least 1 follow-up (30 patients, 67%) were enrolled in the final analysis. The majority of these 30 patients were men (76.7%), had oral cancer (40%), had stage III or IV disease (60%), received surgical intervention (63%) and were treated with chemoradiation (80%). A curative total dose of 66 to 70 Gy was delivered to 23 (76.7%) patients, half of whom received HT. Patients who received chemotherapy had significantly lower global QoL scales (mean difference, 27.94; 95% CI, 9.33-46.55; *p*=0.005). Global QOL, physical function, symptoms of sticky saliva, cough, feelings of illness and weight loss improved significantly between 2 weeks and 3 months. There was no significant difference between 3 and 6 months. Interestingly, improvements in social function, social contact, pain and nutrition reached significance at 6 months. Subgroup analysis revealed greater pain relief over time for patients who underwent HT (*p*=0.030). Moreover, patients who participated in swallowing rehabilitation programs had a greater decrease in nausea and vomiting (*p*=0.036).

**Conclusion:**

HNC patients treated with modern RT techniques experience improved QOL and physical function over time. The most significant improvement occurs between 2 weeks and 3 months, after which the improvement plateaus. However, social function, social contact, pain and nutrition may require longer recovery intervals after treatment. HT with daily image guidance could provide a therapeutic opportunity for improving pain relief in patients with HNC.

## Background

Radiotherapy (RT) contributes to survival and locoregional control for patients with head and neck cancer (HNC) (1). With increasing survival rates and local control in HNC patients, long-term quality of life (QOL) after curative treatment has gradually become a topic of interest (2). Modern RT techniques aim to improve treatment results (3, 4) and treatment-related side effects (5) by delivering highly conformal dose distributions to the target tissue via image-guided techniques and minimizing dosage over normal adjacent structures. Recent literature on intensity-modulated radiotherapy (IMRT) has shown a significant reduction in xerostomia and improved QOL compared to three-dimensional conformal radiotherapy (3DCRT) (6). While studies have shown comparable results in patients treated with volumetric modulated arc therapy (VMAT) versus IMRT, there are still limited reports regarding QOL resulting from the use of arc-based radiation techniques (7–10).

Hammerlid et al. (11) reported the greatest change in health-related quality of life (HRQOL) for HNC patients within the first year after diagnosis and significant deterioration immediately after completing treatment. Despite successful eradication of disease, we observed a certain number of patients with decisional regrets upon the first few follow-up visits, mostly resulting from symptom burdens of dysphagia, pain and speech disturbance. Despite their awareness of side effects, patients still expect a prompt recovery and return to normal daily life at the time of treatment completion. Although most studies have focused on assessing QOL for more than 6 months, Elumalai et al. (12) reported a more rapid return to baseline QOL in HNC patients treated with VMAT-based radiotherapy.

We previously conducted a cross-sectional study to test factors that contribute to QOL and utility in HNC patients who completed treatment after 6 months (13). In the current study, we sought to assess the changes in QOL and utility in patients with HNC within 6 months and to identify factors that may impact QOL outcomes after radiation therapy in a longitudinal setting.

## Materials and methods

### Study design and patient selection

**Figure 1:**
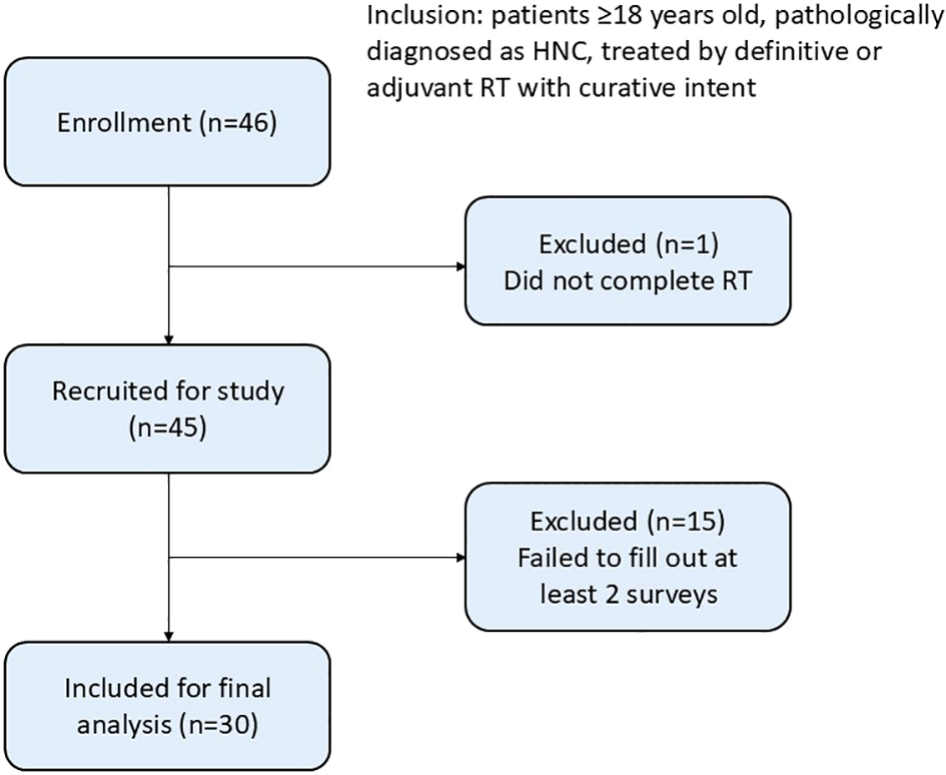
Study design, including inclusion and exclusion criteria.

Patients with HNC who completed treatment between March 2022 and January 2023 were prospectively included in this single-center study. Patients who were more than 18 years old, pathologically diagnosed with HNC, treated with definitive or adjuvant arc-based modern radiotherapy (VMAT or helical tomotherapy, HT) with curative intent and were deemed disease free at the 6-month follow-up were eligible for inclusion. All participants were asked to complete QOL questionnaires, which included the EORTC QLQ-C30, QLQ-H&N 35 and utility score by time trade-off (TTO) at three time points (2 weeks, 3 months and 6 months) after RT completion. Patients with previous treatments, uncontrolled comorbid conditions, developed recurrence or died before the 6-month follow-up were excluded. Participants were followed on an outpatient basis, and those who failed to complete at least two surveys were also excluded (**Figure 1**). This study was approved by the Institutional Review Board of Far Eastern Memorial Hospital (reference number: FEMH 110183-E). Informed consent was obtained from all participants, and the results were analyzed and tabulated.

### Treatment delivery

Radiotherapy delivery protocol for HNC in our institution is standardized according to modalities, namely VMAT and Tomotherapy. For VMAT, treatment is conducted using a linear accelerator (Eleckta Versa HD, Eleckta Oncology Systems Ltd., Crawley, UK), selecting 6 MV photon energy with treatment plans designed via the Pinnacle Treatment Planning System (version 9.8.1, Phillips Healthcare, Andover, MA), including at least two full arcs to ensure conformal dose distribution. For HT, treatment and planning design employs the TomoTherapy Hi Art Planning system (version 5.1.3, Tomotherapy, Inc., Madison, WI, USA). Prior to each HT treatment session, patient positioning and image guidance via MVCT is performed, with daily image adjustments ensuring high precision for treatment localization. Therefore, we create a 3 mm expansion from CTV to PTV considering HT planning, compared to a 5 mm expansion when using VMAT.

### QOL questionnaires

The validated Taiwan Chinese version of the European Organization for Research and Treatment of Cancer Quality of Life Questionnaire-Core 30 (EORTC QLQ-C30) version 3 and the QLQ-H&N35 were used (13). Patients were asked to complete a form including content from the abovementioned questionnaires and 2 additional questions for utility assessment at 3 regular follow-up visits at 2 weeks, 3 months and 6 months after completion of the RT course. The scores of the QLQ-C30 and QLQ-H&N35 items are linearly transformed to scales from 0 to 100. For the functioning scales and global QOL scales, higher scores correlate with better levels of daily functioning. In contrast, for symptom scales, higher scores represent higher levels of symptoms or problems (14).

### EORTC QLQ-C30 version 3

The QLQ-C30 is composed of both multi-item scales and single-item measures, including five functional scales, three symptom scales, a global health status/QOL scale, and six single items. All the scales range from 0 to 100. A high score on the functional scale represents a high level of functioning, and a high score on the symptom scale represents a high level of symptomatology. A high score on the global QOL represents a high general QOL. The manual contains scoring procedures for the QLQ-C30 version 3.0 and QLQ-C30 version 3.0, which are used in the current studies. All scales were scored in accordance with the EORTC scoring manual (15).

### EORTC QLQ-H&N35

The QLQ-H&N35 is a module used for assessing QOL specifically in HNC patients. The QLQ-H&N35 included seven multiple-item scales and six single-item scales (13). The seven multiple-item scales assess the symptoms of pain, swallowing ability, sensation (taste/smell), speech, social eating, social contact, and sexuality. Six single-item scales survey the presence of symptomatic problems associated with the teeth, mouth opening, dry mouth (xerostomia), sticky saliva, coughing, and malaise. A high score on the symptom scale represents a high level of symptomatology.

### Utility instrument

The time trade-off (TTO) has previously been used to assess laryngeal utility in several studies (16). We used TTO instead of EuroQol-5D (EQ-5D) as our measurement technique for HNC survivors given that TTO is a “choice task” rather than a “rating task”, while the latter are prone to scaling bias. TTO is recommended when performing cost-utility analysis using quality-adjusted life years as an outcome. The patients were first asked to imagine how many years they had left to live (X). Then, they could choose to give up some life years (Y) to live for a shorter period in perfect health. The utility would then be (X-Y)/X, according to the TTO method. The values are anchored at 1 (full health) and 0 (dead); higher values indicate greater health utility. Utility and QOL were assessed simultaneously in our study.

### Statistical analysis

Patient characteristics were summarized with descriptive statistics. Categorical variables are presented as frequencies and percentages, whereas continuous variables are expressed as means with standard deviations (SDs). Linear mixed models with random intercepts for participants and restricted maximum likelihood estimates were used to assess the effects of variables (age, sex, primary site, cancer stage, treatment, radiation dose and radiation method). Participants with at least 1 follow-up measurement to examine changes in QOL and utility outcomes were enrolled for evaluation. In each model, the interaction between time (visit) and study variables was tested, while marginal *post hoc* estimates were calculated to test the association of variables with each outcome by visit. Overall changes in QOL and utility scores at different time points were tested using paired t tests.

All analyses were carried out in SPSS, version 21.0 (IBM, Armonk, NY, USA) and RStudio, version 4.0.3 (PBC, Boston, MA, USA). Two-tailed p values <0.05 were considered to indicate statistical significance.

## Results

### Patient characteristics

Of the 45 patients (46 patients who met the inclusion criteria were approached, and 45 [97.8%] patients were enrolled after they provided informed consent) recruited from March 2022 to January 2023, 30 (67%) patients completed the surveys at 2 weeks with at least 1 follow-up.

The demographic and clinical characteristics of the patients are shown in **Table 1**. Among the 30 patients included in the final analysis, 12 (40%) had oral cancer, 6 (20%) had nasopharyngeal carcinoma, 2 (6.7%) had oropharyngeal cancer, 5 (16.7%) had hypopharyngeal/laryngeal cancer, and 5 (16.7%) had cancer of other head and neck origins. The major characteristics of the patients in the study group were as follows: adult males (76.7%), stage III or IV disease (60%), surgical intervention (63%) and treatment with chemoradiation therapy (CCRT, 80%). In total, 66 to 70 Gy was delivered to 23 (76.7%) patients with curative intent, and half of them were treated with HT. Meanwhile, there is a higher proportion of patients with more advanced stage III or IV disease (81.3% vs. 35.7%) and received chemotherapy (93.8% vs. 64.3%) in the HT group compared to VMAT group.

**Table 1:** Characteristics of head and neck cancer patients enrolled for final analysis.

Participants (n=30)	No. (%)
***Age, median (range), years***	56 (27-80)
***Gender***	
Male	23 (76.7)
Female	7 (23.3)
***Primary site***	
Oral cavity	12 (40.0)
Nasopharynx	6 (20.0)
Oropharynx	2 (6.7)
Larynx/ Hypopharynx	5 (16.7)
Other	5 (16.7)
***Cancer stage (AJCC 8^th^)***	
I-II	12 (40.0)
III-IV	18 (60.0)
***Treatment category***	
Surgery plus adjuvant	19 (63.3)
CCRT	15 (50.0)
RT only	4 (13.3)
Nonsurgical	11 (36.7)
CCRT	9 (30.0)
RT only	2 (6.7)
***RT dose***	
6600 ~ 7000 cGy	23 (76.7)
rmsp6000 ~ 6400 cGy	7 (23.3)
***Techniques applied for radiotherapy***	
rmspVMAT	14 (46.7)
rmsp HT	16 (53.3)
***Swallow rehabilitation***	13 (43.3)
***Patient services***	27 (90.0)

CCRT, concurrent chemoradiation therapy; HT, helical tomotherapy; RT, radiotherapy; VMAT, volumetric modulated arc therapy.

### Factors associated with overall global QOL and utility

Chemotherapy and time after completion of therapy were predictive factors for overall global QOL. The mean global health status scale at 2 weeks after the completion of radiotherapy was 45.28 out of 100 for the 30 patients included in the analysis: 43.06 for oral cancer, 52.78 for NPC, 16.71 for oropharyngeal cancer, 63.33 for hypopharyngeal/laryngeal cancer, and 35 for other cancers (p = 0.373, one-way ANOVA). The overall mean utility index at 2 weeks after the completion of radiotherapy was 0.42 out of 1. The mean utility indices were 0.47 for oral cancer, 0.44 for NPC, 0.25 for oropharyngeal cancer, 0.36 for hypopharyngeal/laryngeal cancer, and 0.40 for thyroid cancer (p = 0.603, one-way ANOVA). According to our mixed model analysis, the global QOL was significantly lower in patients who received chemotherapy (mean difference, 27.94; 95% CI, 9.33-46.55; *p*=0.005). Aside from the effect of chemotherapy, the effect of time on QOL improvement was also prominent (mean difference, 9.71; 95% CI, 3.88-15.54, *p*<0.001), while no obvious difference was observed in the other remaining subgroups (**Table 2**). The effects of chemotherapy and time were not significant in the utility domain (**Table 2**).

**Table 2:** Comparison of global QOL, utility using multiple variables.

	*Global QOL*			*Utility*	
	Mean (SD)		P value	Mean (SD)	P value
***Age***					
≤ 55 y/o	51.82 (25.20)	.310		0.35 (0.37)	.304
> 55 y/o	61.12 (29.50)			0.48 (0.36)	
***Gender***					
Male	54.46 (26.68)	.275		0.43 (0.39)	.954
Female	65.09 (30.79)			0.42 (0.30)	
***Tumor site***					
Oral cavity	52.87 (22.96)	.250		0.49 (0.39)	.850
Nasopharynx	59.52 (31.50)			0.48 (0.27)	
Oropharynx	33.33 (18.26)			0.13 (0.25)	
Larynx/ Hypopharynx	72.43 (28.05)			0.44 (0.38)	
Other	54.17 (28.54)			0.30 (0.40)	
***Cancer stage***					
I-II	63.13 (26.80)	.259		0.40 (0.33)	.638
III-IV	52.71 (28.27)			0.45 (0.40)	
***Chemotherapy***					
Yes	50.57 (26.53)	.005*		0.45 (0.38)	.503
No	79.61 (20.36)			0.34 (0.30)	
***Operation***					
Yes	54.34 (26.41)	.574		0.42 (0.39)	.981
No	62.16 (30.38)			0.44 (0.34)	
***RT dose***					
6600 ~ 7000 cGy	53.94 (29.37)	.114		0.40 (0.35)	.462
6000 ~ 6400 cGy	68.14 (19.37)			0.51 (0.42)	
***RT method***					
VMAT	64.68 (25.57)	.123		0.35 (0.33)	.205
HT	50.21 (28.58)			0.51 (0.40)	
***Swallowing rehabilitation***					
Yes	47.66 (26.20)	.106		0.44 (0.37)	.868
No	64.22 (27.39)			0.42 (0.37)	
***Time***					
2 weeks post-RT	45.28 (28.67)	<.001*		0.42 (0.07)	.726
3 months post-RT	66.74 (24.48)			0.40 (0.07)	
ermsp6 months post-RT	63.48 (25.43)			0.44 (0.08)	

### Changes in QOL and symptom burden

Global QOL, physical function, swallowing function, sticky saliva, cough, malaise and weight loss improved significantly between 2 weeks and 3 months after RT. No significant difference between any of the scales at 3 and 6 months was observed. Interestingly, the improvements in social function, social contact, speech, pain and nutrition were non-significant at 3 months but still reached significance at 6 months when compared to the scales at 2 weeks (**Table 3**).

**Table 3:** Changes in QOL, utility and symptom burdens at different time points.

Paired t-test	2 weeks - 3 months			2 weeks - 6 months			3months - 6 months		
	n	Mean difference	P value	n	Mean difference	P value	n	Mean difference	P value
Global QOL	22	-20.152	<.001*	23	-22.536	.001*	15	2.778	.173
Utility	18	0.044	.521	18	-0.019	.790	14	-0.022	.314
Physical	22	-7.273	.026*	23	-7.246	.049*	15	2.667	.054
Role	22	0	1	23	-1.449	.740	15	2.222	.334
Emotional	22	-6.061	.172	23	-5.797	.122	15	0.556	.334
Cognitive	22	-2.273	.613	23	-0.725	.833	15	4.444	.164
Social	22	-6.818	.303	23	-12.319	.010*	15	2.222	.334
Fatigue	22	5.556	.352	23	3.382	.307	15	-5.185	.404
Nausea & vomiting	22	5.303	.425	23	9.420	.067	15	2.222	.334
Pain	22	12.879	.077	23	10.870	.018*	15	-3.333	.334
Dyspnea	22	1.515	.771	23	4.348	.328	15	2.222	.334
Insomnia	22	1.515	.747	23	-1.449	.770	15	-6.667	.384
Appetite loss	22	7.576	.204	23	2.899	.539	15	-4.444	.334
Constipation	22	6.061	.257	23	5.797	.213	15	0	1
Diarrhea	22	-4.545	.329	23	-7.246	.171	15	-8.889	.104
Financial difficulties	22	0	1	23	4.348	.418	15	0	1
Swallow	22	18.939	.002*	23	19.203	.002*	15	-5.556	.406
Sense	22	3.030	.623	23	4.348	.366	15	-3.333	.582
Speech	22	9.596	.092	23	13.043	.021*	15	-2.963	.217
Social eating	22	6.061	.242	23	9.420	.062	15	-3.333	.486
Social contact	22	6.061	.304	23	11.304	.015*	15	-1.333	.334
Sexuality	22	5.303	.432	23	-1.449	.817	15	-10.000	.167
Teeth	22	9.091	.162	23	4.348	.451	15	-13.333	.111
Open mouth	22	4.545	.451	23	7.246	.233	15	-4.444	.334
Dry mouth	22	10.606	.148	23	5.797	.295	15	-6.667	.189
Saliva	22	24.242	<.001*	23	17.391	.001*	15	-6.667	.384
Cough	22	15.152	.002*	23	8.696	.186	15	-4.444	.546
Ill	22	13.636	.025*	23	13.043	.009*	15	0	1
Painkiller	22	22.727	.057	23	34.783	.002*	15	0	1
Nutrition	22	18.182	.162	23	26.087	.011*	15	20.000	.082
NG	22	0	1	23	4.348	.575	15	0	1
Weight loss	22	36.364	.002*	23	30.435	.016*	15	-13.333	.164
Weight gain	22	-9.091	.329	23	-8.696	.426	15	0	1

### Factors associated with changes in global QOL and symptom burdens

**Figure 2:**
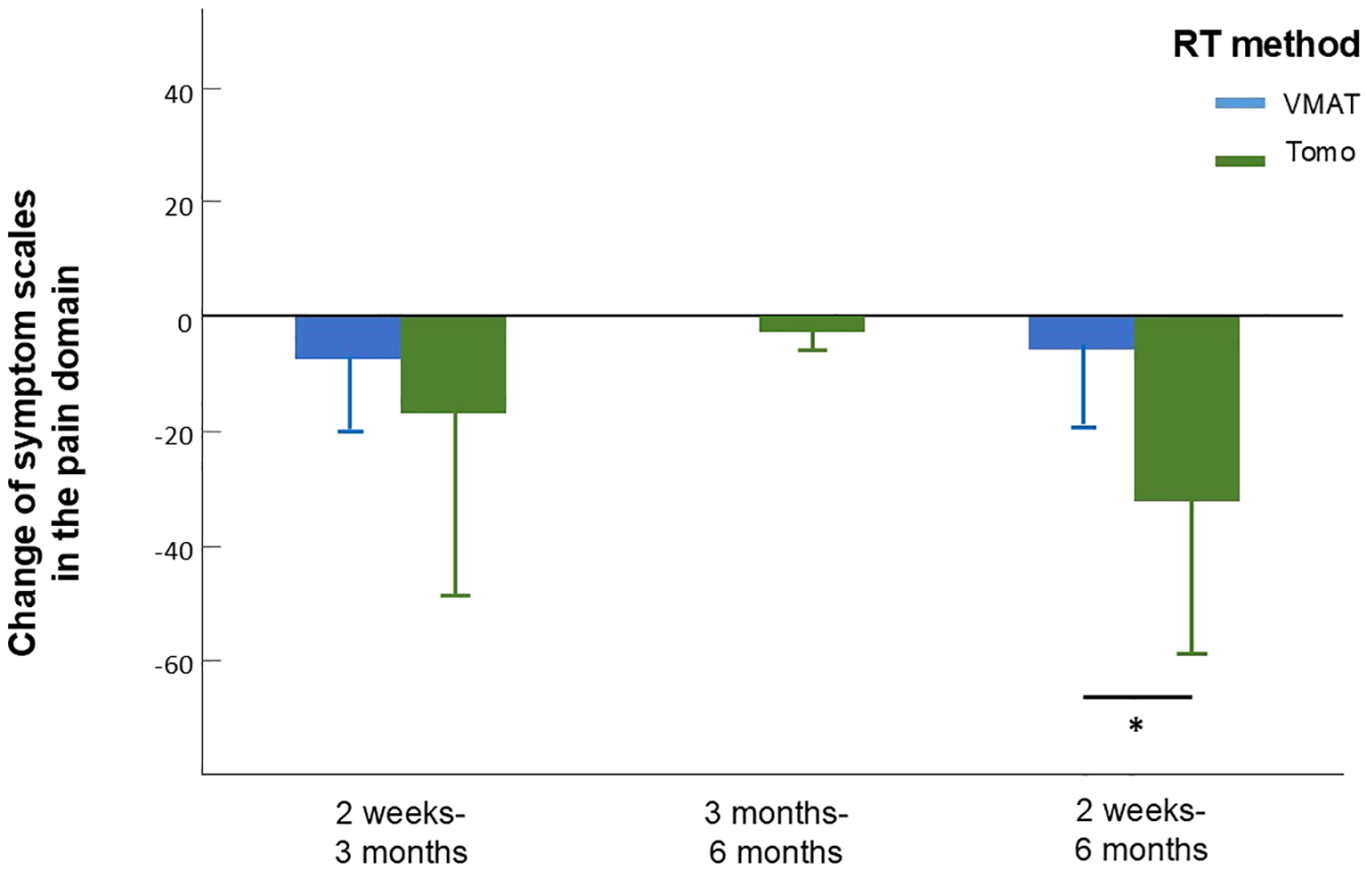
Change of symptom scales in the pain domain in patients who received treatment applying VMAT or tomotherapy assessed between different time intervals. Between 2 weeks to 6 months, patients who received tomotherapy experienced greater pain relief assessed by the reduce of EORTC QLQ-H&N35 pain scale. Error bars represent standard deviation (SD).

**Figure 3:**
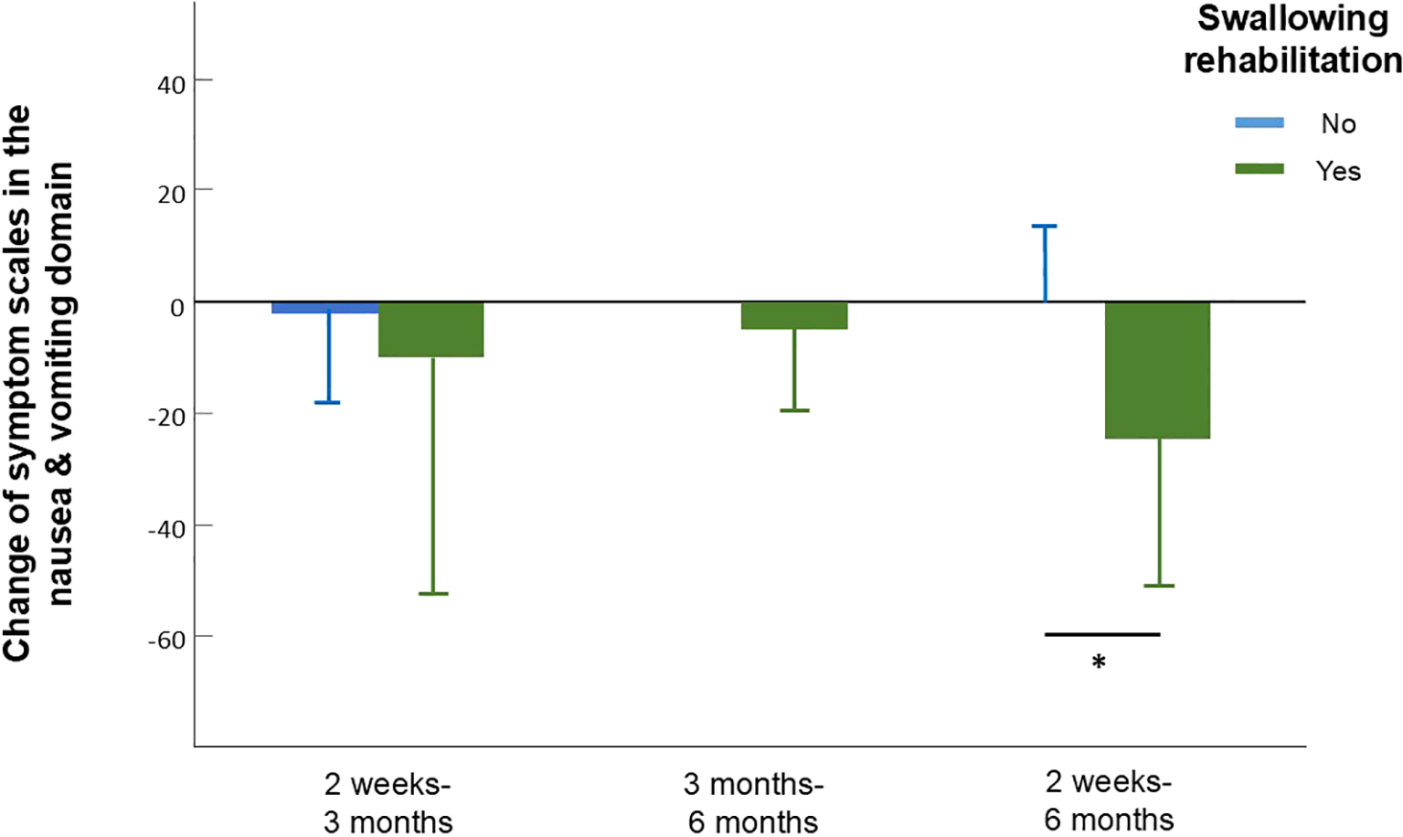
Change of symptom scales in the nausea and vomiting domain in patients who did and did not participate in swallowing rehabilitation programs assessed between different time intervals. Between 2 weeks to 6 months, patients who participated in swallowing rehabilitation programs had a more obvious decrease in symptoms of nausea and vomiting assessed by the reduce of EORTC QLQ-C30 nausea and vomiting scale. Error bars represent standard deviation (SD).

We analyzed values of QOL and symptom burden change separately according to different factors including age, gender, treatment, radiation dose, and radiation method. Due to the significant effect of chemotherapy on global QOL, we did not include chemotherapy as part of our subgroup analysis. According to the subgroup analysis, patients treated with helical tomotherapy (HT) experienced greater pain relief over time than did those treated with VMAT (mean difference, 21.78; 95% CI, 2.27-41.29; *p*=0.03, **Figure 2**, **Supplementary Table S1.1**). At 6 months after treatment, the group that received a lower RT dose (60-64 Gy) showed greater improvement in cognitive function (*p*=0.039) and sensory problems (*p*=0.030) than did the group treated with a higher RT dose (66-70 Gy). Patients who underwent surgery showed more significant improvements in cognitive function (*p*=0.048), emotion (*p*=0.023), pain (*p*=0.046) and swallowing difficulty (*p*=0.017). Moreover, patients who participated in swallowing rehabilitation programs had more obvious baseline symptoms and experienced more significant decreases in nausea and vomiting (mean difference, 24.07; 95% CI, 1.93-46.22; *p*=0.036, **Figure 3**, **Supplementary Table S1.1**).

### Longitudinal analysis on patients with data at all three time points

Among the 30 patients analyzed, only 15 had complete data at 2 weeks, 3 months, and 6 months. To validate our results as much as possible, we also conducted additional analysis focusing on the 15 patients with data at all three time points.

Among the 15 patients, the majority was male (66.7%), underwent operation (66.7%) and received a higher dose of RT of 66 to 70 Gy (80%). In 8 (53.3%) of the patients, VMAT was applied while the other 7 (46.7%) patients were treated with tomotherapy. 6 (40%) patients participated in swallowing rehabilitation. Chemotherapy and time after completion of therapy were repeatably predictive factors for overall global QOL.

**Figure 4:**
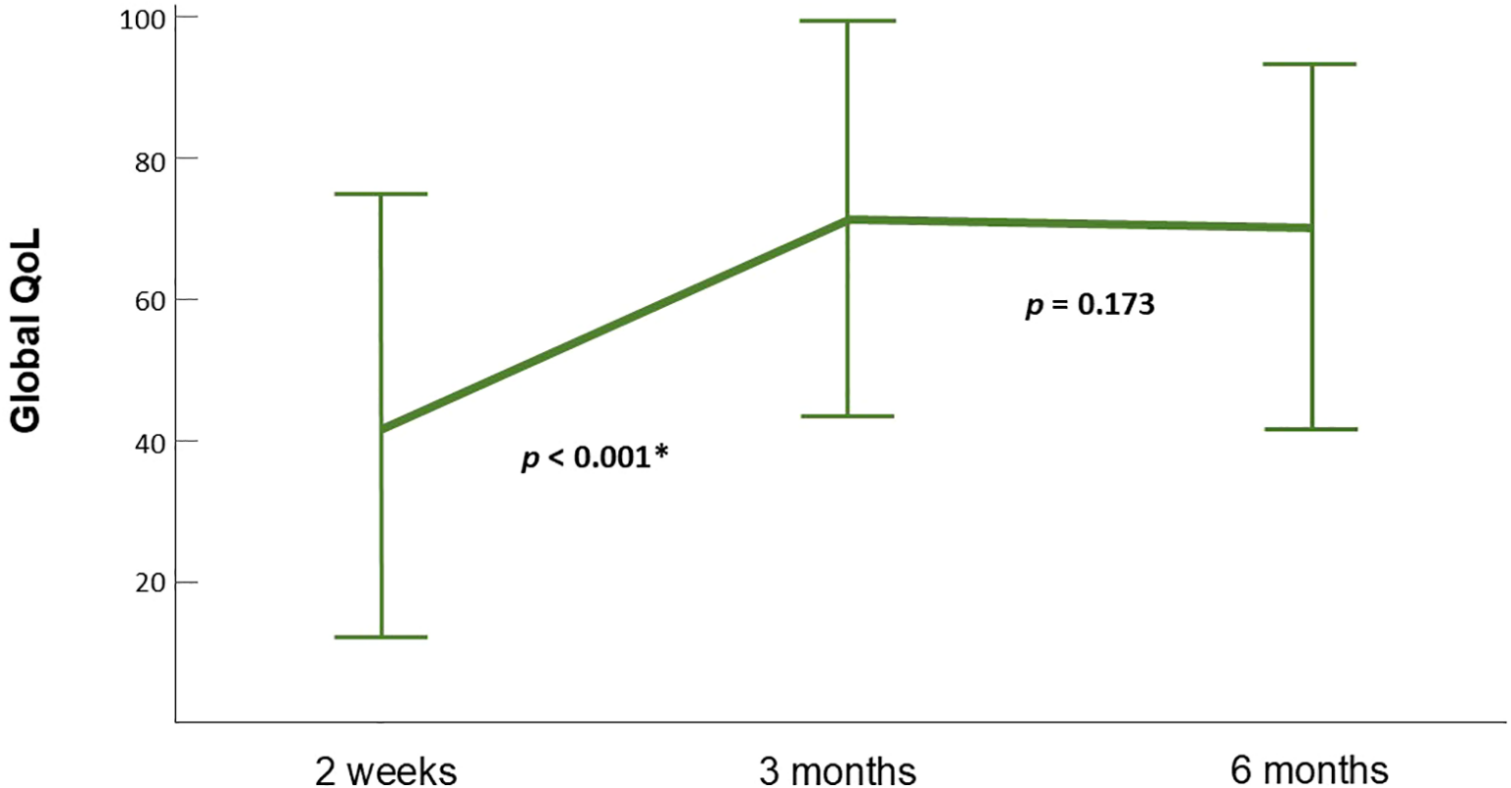
Dynamic change of global QOL scales. Global QOL improved significantly between 2 weeks and 3 months and reached a plateau between 3 and 6 months. The figure is illustrated according to patients that contain data at all time points (n=15). Error bars represent standard deviation (SD).

Global QOL, physical function, social function, fatigue, pain, swallowing function, speech, sticky saliva, cough, malaise and weight loss improved significantly between 2 weeks and 3 months after RT. No significant difference between any of the scales at 3 and 6 months was observed (**Figure 4**). Improvements in social contact, painkiller use and nutrition were non-significant at 3 months but reached significance at 6 months when compared to the scales at 2 weeks. (**Supplementary Table S2**)

In subgroup analysis of factors effecting QOL change, patients treated with HT experienced greater pain relief over time compared to VMAT but failed to reach statistical significance (*p*=0.087, **Supplementary Table S1.2**). Patients who participated in swallowing rehabilitation programs experienced more significant decreases in nausea and vomiting (*p*=0.013, **Supplementary Table S1.2**).

## Discussion

This is a novel study that longitudinally evaluated global QOL, function, symptom scales and utility in Asian HNC patients. We found that HNC survivors treated with modern RT techniques experience post-treatment improvements in QOL and physical function over time.

Compared to 3DCRT, a significantly shorter duration of feeding tube placement has been reported in HNC patients who receive IMRT (17). Vergeer et al. (18) also demonstrated statistically significant reductions in xerostomia with IMRT compared with 3DCRT. At 6 months post-treatment, 67% and 41% of patients treated with 3DCRT and IMRT, respectively, reported moderate or severe xerostomia. Moreover, IMRT minimizes radiation to surrounding tissues, which possibly results in a better outcome in multiple QOL domains in comparison with conventional RT (18–20). These data suggest that significant QOL benefits are gained by applying IMRT in patients with HNC.

The University of Michigan designed a prospective study of oropharyngeal carcinoma patients treated by IMRT with specific sparing of uninvolved swallowing organs, which resulted in absent to minimal dysphagia (21, 22). Without specific sparing of swallowing structures, however, oropharyngeal carcinoma patients treated with IMRT demonstrated a 7% rate of feeding-tube dependence at 1 year (23). These results indicated that targeted sparing of swallowing structures may provide additional benefits in preventing long-term dysphagia and improving QOL.

A growing body of evidence shows that VMAT is superior or noninferior to IMRT in terms of dosimetry (8, 24). While previous literature reported a return of QOL to baseline at 6-12 months in HNC patients treated with IMRT (11, 25–30), in our current study, when VMAT or HT was applied, the global QOL reached a plateau at 3 months after the completion of RT, regardless of age, sex, cancer site, stage, treatment method or total radiation dose. Loorents et al. (31) reported that most symptoms and functions deteriorated significantly by the end of RT for HNC patients, improved gradually by 3 months and reached baseline levels at 12 months after RT completion. In a study conducted by Periasamy et al., the QOL in oropharyngeal, laryngeal and hypopharyngeal patients treated with VMAT returned to baseline values by 3 months post-treatment, which is consistent with our results (32). The accelerated symptom recovery after RT may be due to the greater dose-sparing effect achieved by the VMAT technique or to advancements in medical treatment, such as new medications that prevent or alleviate the side effects of cancer treatment (33, 34).

Over time, patients who underwent surgery had a more significant improvement in swallowing and pain, which are likely the initial consequences of postoperative inflammation and nerve overstimulation (35, 36). A meta-analysis of 82 studies evaluating pain in HNC patients treated according to various schemes estimated that pain occurred in 57% of patients before treatment and in 42% of patients after treatment (37). In the present study, the improvement in pain over time was more significant in patients treated with helical tomotherapy (HT) than in patients treated with VMAT. Although the degree of pain relief between the two groups was comparable between 2 weeks and 3 months, patients in the HT group had continue improvement on pain between 3 months and 6 months while patients in the VMAT group seemed to remain constant. Therefore, despite having higher pain scales at baseline, HT group patients managed to reach almost equal results as VMAT group at 6 months. Compared with those of VMAT and IMRT, better conformation numbers, healthy tissue conformity indices and homogeneity have been achieved by HT in HNC patients (38). Moreover, image-guided radiotherapy (IG IGRT) is associated with significantly greater overall survival and locoregional survival and lower toxicity after daily position correction than is non-IG IMRT (5). For HNC patients treated with HT in our facility, daily image guidance techniques are routinely applied with a lesser margin to form the planning target volume (PTV), which could result in a smaller treatment volume and decreased toxicity. The results from the abovementioned IMRT and VMAT studies as well as our current study indicate that IGRT, such as HT, may provide more QOL-related benefits than IMRT and VMAT. Longer follow up will be needed to support our point of view.

The Radiation Therapy Oncology Group and the Head and Neck Intergroup (RTOG 91-11) confirmed that patients receiving radiotherapy with chemotherapy had greater chemotherapy-related toxic effects and increased rates of severe radiation-related toxic effects (39). According to Barker et al. (40), HNC patients who were asymptomatic at baseline reported a prompt worsening of QOL following CCRT. Growing evidence also shows a trend toward worse QOL in patients receiving combined chemoradiotherapy (CCRT) than in those receiving RT alone (33, 41–43). Similarly, the QOL and symptom-related scales were worse in patients who received chemotherapy in our study. Further studies should focus on new chemotherapy regimens and more precise RT modalities to decrease side effects and improve QOL with equal clinical effects.

Swallowing dysfunction after HNC therapy contributes to reduced patient QOL, increased morbidity, and increased mortality. Goepfert et al. (27) reported that the decisional regrets of oropharyngeal cancer patients were mainly associated with symptom burdens focused on swallowing difficulty, depression and pain. The incidence of aspiration for liquids in patients with oropharyngeal cancer treated with CCRT was 24% at 12 months (22) and 7% at 6 months for those treated with accelerated radiotherapy (44). Patterson et al. (45) reported that there was a significant reduction in swallowing scores for HNC patients treated with CCRT from pretreatment to 3 months posttreatment and no improvement in scores from 3 to 12 months post-CCRT. Notably, earlier intervention potentially helped achieve better responses in terms of diet and QOL. Van Daele et al. (46) reported that starting a swallowing therapy program within one year of RT completion of improved QOL and diet performance to a greater extent than did starting such a program later. Other studies have shown that the addition of swallowing rehabilitation to the post-radiation period for a longer duration may further benefit swallowing outcomes, particularly in patients with negative predictors (46–49). In our study, patients who received early rehabilitation intervention had more obvious baseline symptoms. Although they did not seem to show significant improvement in swallowing dysfunction, they had a more obvious decrease in symptoms of nausea and vomiting (**Figure 4**, *p*=0.036), which potentially reduces the risk and severity of liquid aspiration. During follow up period, 6 of our patients developed aspiration-related events; 3 of them had participated in swallowing rehabilitation programs and 3 did not. Upon further investigation, we found that the 3 patients which received swallowing rehabilitation programs but still developed aspiration-related events had advanced stage disease and primary site of oropharynx or hypopharynx. To be noted, all of them recovered after medical treatment. The other 3 that developed aspiration-related events which did not participate in swallowing rehabilitation programs before were oral cancer patients and 2 of them had early-stage disease. One of them with advanced stage disease eventually failed to recover after aspiration-related event.

Although Nallani et al. (50) reported a positive correlation between anxiety and decision regret at the 3-month follow-up and lasting to the 6-month follow-up, a low incidence of emotional problems was noted at 2 weeks and at 3 and 6 months posttreatment in our study. As women with HNC tend to have more emotional problems than men do according to previous literature (51), we speculate that the low incidence of emotional problems observed may have been because there were more men (77%) included in our study, which is consistent with results reported by Loorents et al. (31)

Furthermore, there was no difference in the QOL change between patients who did and did not receive patient services (including psychologist, nutritionist and social worker consultations). However, we still believe that such services play an important role in social function and the long-term recovery of nutritional status because 90% of our patients had patient service records.

In our additional analysis focusing on the 15 patients with data at all three time points, the characteristics were similar and had the same predictive factors for overall global QOL. The trend of dynamic change was consistent with our original group, showing significant improvement of global QOL, physical function and certain symptom burdens between 2 weeks and 3 months after RT followed by a plateau from 3 months to 6 months. Although improvement of social function and pain were also detected at 3 months in this group, the significance was increased at 6 months while significant improvements of social contact, painkiller use and nutrition were only reached at 6 months after RT. The advantage of HT over VMAT on pain relief was still observed but did not reach statistical significance in this group presumably due to an even smaller sample size.

Our current results should be interpreted cautiously in light of several limitations. First, despite the prospective nature of our study, the sample size included in the final analysis was small (n = 30) and only half of which had complete data at all three time points. Thus, the generalizability of the research results is limited. In our previous study published in 2019 (13), we analyzed various factors associated with QOL of head and neck cancer survivors 6 months after completing definitive treatment including education level, marriage status, socioeconomic class and occupation status. Lower annual family income was found to be associated with generally lower QOL and utility scores. However, the trend was not found in this longitudinal study which may also be relevant to our relatively smaller sample size. Second, our baseline was set at 2 weeks after the last course of radiation therapy. Therefore, we did not collect questionnaires from these patients before treatment delivery. Third, although the samples were all obtained from HNC survivors, the various cancers included oral cancer, oropharyngeal cancer, nasopharyngeal cancer, hypopharyngeal cancer, and laryngeal cancer. In addition, some patients needed to undergo surgery prior to CCRT, and some did not. Therefore, the symptoms or side effects might not be the same and could affect the prediction of QOL changes. Therefore, the results need further validation from larger longitudinal investigations, and it is recommended that future studies enroll patients diagnosed with a specific type of HNC to increase the homogeneity. In a practical point of view, the original design of EORTC questionnaires is precise and comprehensive but contain numerous items which may interfere with subject’s attention and compliance. Therefore, we will consider focusing on questions limited to a certain function domain or symptom burden in the future to ensure better compliance and data integrity during longitudinal analysis.

## Conclusion

HNC survivors treated with modern RT techniques experience improvements in QOL and physical function over time. The most significant improvement occurs between 2 weeks and 3 months, after which it substantially reaches a plateau. Social function, social contact, pain and nutrition may require longer recovery intervals after treatment. HT with daily image guidance could provide a therapeutic opportunity for improving pain relief in patients with HNC. Further replicative results could eventually guide us toward better clinical management.

## Data Availability

The raw data supporting the conclusions of this article will be made available by the authors, without undue reservation.
