# Supplementary material for: Improved quality of life in head and neck cancer patients treated with modern arc radiotherapy techniques – A prospective longitudinal analysis
Source: Front Oncol. 2024 Sep 23;14:1424034. doi: 10.3389/fonc.2024.1424034 (PMC11456567; doi:10.3389/fonc.2024.1424034)
Supplement: Supplementary file 1 [file Table1.docx]

**Table S1.1**: Factors effecting change of QoL and symptom burdens (n=30)

|  |  | Age | | Gender | | Operation | | RT dose | | RT method | | Swallowing rehabilitation | |
| --- | --- | --- | --- | --- | --- | --- | --- | --- | --- | --- | --- | --- | --- |
|  | Time interval | p | Mean Difference | p | Mean Difference | p | Mean Difference | p | Mean Difference | p | Mean Difference | p | Mean Difference |
| Global | 1-2 | 0.843 | 2.12091 | 0.594 | -4.76238 | 0.223 | -13.33179 | 0.404 | 10.58671 | 0.821 | 2.42455 | 0.487 | 7.17864 |
| Global | 2-3 | 0.185 | -6.94500 | 0.437 | -3.33300 | 0.437 | -3.33300 | 0.494 | 3.47250 | 0.716 | 1.48768 | 0.577 | 2.31444 |
| Global | 1-3 | 0.983 | 0.27833 | 0.095 | -21.33500 | 0.395 | -11.33300 | 0.211 | 19.30667 | 0.865 | -2.17250 | 0.846 | -2.52619 |
| Utility | 1-2 | 0.578 | -0.07846 | 0.311 | 0.14995 | 0.226 | 0.15807 | **0.029*** | -0.38537 | 0.830 | -0.03057 | 0.196 | -0.18307 |
| Utility | 2-3 | 0.455 | -0.040079 | 0.229 | 0.065972 | 0.753 | 0.017063 | 0.768 | -0.028535 | 0.229 | -0.065972 | 0.256 | -0.052407 |
| Utility | 1-3 | 0.494 | -0.14087 | 0.616 | 0.10861 | 0.693 | 0.08198 | 0.163 | -0.49389 | 0.473 | -0.15444 | 0.256 | -0.17241 |
| Physical | 1-2 | 0.847 | 1.21273 | 0.072 | 11.68248 | 0.244 | -7.50054 | 0.290 | 7.84518 | 1.000 | -0.00182 | 0.110 | 13.23041 |
| Physical | 2-3 | 0.684 | -1.11111 | 0.114 | -5.99900 | 0.478 | -2.00000 | 0.053 | 3.33333 | 0.448 | 2.14107 | 0.357 | 2.96111 |
| Physical | 1-3 | 0.677 | 4.81500 | 0.675 | 5.33500 | 0.504 | -6.66800 | 0.157 | 16.11333 | 0.773 | -2.74143 | 0.335 | 7.03563 |
| Role | 1-2 | 0.497 | 9.09182 | 0.136 | 20.95095 | 0.149 | -19.64214 | 0.169 | 21.56835 | 0.652 | 6.06091 | 0.146 | 22.43631 |
| Role | 2-3 | 0.363 | -5.55500 | 0.374 | -6.66600 | 0.374 | -6.66600 | 0.635 | 2.77750 | 0.356 | 4.76143 | 0.363 | 5.55500 |
| Role | 1-3 | 0.897 | -1.85111 | 0.571 | 8.33300 | 0.425 | -11.66500 | 0.112 | 26.38667 | 0.865 | -2.38000 | 0.551 | 5.42365 |
| Emotional | 1-2 | 0.865 | -1.51455 | 0.137 | 13.81048 | 0.070 | -16.07321 | 0.941 | 0.78565 | 0.613 | -4.54545 | 0.835 | 2.11590 |
| Emotional | 2-3 | 0.435 | 0.92556 | 0.374 | -1.66600 | 0.500 | 0.83300 | 0.635 | 0.69417 | 0.356 | 1.19000 | 0.363 | 1.38833 |
| Emotional | 1-3 | 0.640 | -5.09333 | 0.414 | 9.16700 | **0.023*** | -13.68292 | 0.877 | -2.08250 | 0.451 | -8.78071 | 0.652 | -4.16619 |
| Cognitive | 1-2 | 0.240 | -10.60545 | 0.141 | 14.12838 | 0.069 | -16.66732 | 0.181 | 14.31459 | 0.620 | 4.54364 | 0.253 | 11.92190 |
| Cognitive | 2-3 | 0.169 | 7.40778 | 0.178 | -13.33400 | 0.622 | -3.33200 | 0.484 | 5.55583 | 0.926 | 0.59661 | 0.777 | 1.85333 |
| Cognitive | 1-3 | 0.319 | -11.11056 | 0.777 | 3.33400 | **0.048*** | -13.88783 | **0.039*** | 26.39000 | 0.852 | 2.08107 | 0.555 | 4.23183 |
| Social | 1-2 | 0.910 | 1.51545 | 0.309 | 14.44371 | 0.309 | -13.98804 | 0.596 | 8.43282 | 0.911 | -1.51545 | 0.860 | 2.30810 |
| Social | 2-3 | 0.435 | 3.70333 | 0.374 | -6.66600 | 0.500 | 3.33300 | 0.635 | 2.77750 | 0.356 | 4.76143 | 0.363 | 5.55500 |
| Social | 1-3 | 0.737 | 3.70278 | 0.771 | 3.33400 | 0.884 | -1.66700 | 0.203 | 16.66667 | 0.212 | -13.09464 | 0.909 | -1.05794 |
| Fatigue | 1-2 | 0.558 | 7.07000 | 0.116 | -19.79133 | 0.291 | 13.09732 | 0.921 | -1.43918 | 0.676 | -5.05000 | 0.994 | -0.08666 |
| Fatigue | 2-3 | 0.359 | -11.72833 | 0.348 | 18.88900 | 0.562 | -7.77800 | 0.683 | -6.48167 | 0.311 | -14.08732 | 0.329 | -16.04944 |
| Fatigue | 1-3 | 0.847 | 1.85000 | 0.306 | 9.99700 | 0.500 | -6.66500 | 0.379 | -10.18750 | 0.704 | -3.56964 | 0.414 | -5.55571 |
| Nausea & vomiting | 1-2 | 0.911 | -1.51545 | 0.669 | -6.19171 | 0.116 | 21.42893 | 0.875 | 2.54694 | 0.574 | 7.57727 | 0.445 | 9.99997 |
| Nausea & vomiting | 2-3 | 0.363 | -5.55500 | 0.374 | -6.66600 | 0.374 | -6.66600 | 0.635 | 2.77750 | 0.356 | 4.76143 | 0.363 | 5.55500 |
| Nausea & vomiting | 1-3 | 0.907 | 1.85167 | 0.920 | -1.66700 | 0.612 | 8.33500 | 0.427 | 15.27417 | 0.074 | 26.19143 | **0.036*** | 24.07333 |
| Pain | 1-2 | 0.075 | 24.24273 | 0.215 | -24.28486 | **0.046*** | 27.25034 | 0.754 | -5.09753 | 0.818 | -3.21949 | 0.702 | 5.38459 |
| Pain | 2-3 | 0.363 | -1.39000 | 0.374 | -1.66800 | 0.374 | -1.66800 | 0.635 | 0.69500 | 0.356 | 1.19143 | 0.363 | 1.39000 |
| Pain | 1-3 | 0.188 | 18.98000 | 0.414 | -12.50000 | 0.324 | 15.00100 | 0.294 | -18.74833 | **0.030*** | 21.78053 | 0.398 | 9.12944 |
| Dyspnea | 1-2 | 0.389 | -9.09091 | 0.676 | -4.76076 | 0.830 | 2.38143 | 0.400 | 10.58776 | 0.777 | 3.03091 | 0.264 | 11.79495 |
| Dyspnea | 2-3 | 0.363 | -5.55500 | 0.500 | 3.33300 | 0.500 | 3.33300 | 0.635 | 2.77750 | 0.351 | -4.16625 | 0.435 | -3.70333 |
| Dyspnea | 1-3 | 0.070 | -22.22167 | 0.806 | 3.33400 | 0.806 | 3.33400 | 0.484 | 11.11083 | 0.460 | 10.11946 | 0.303 | 11.11183 |
| Insomnia | 1-2 | 0.753 | -3.03182 | 0.248 | -11.74486 | 0.741 | -4.16625 | 0.351 | 10.58718 | 0.753 | -3.03000 | 0.545 | 5.89710 |
| Insomnia | 2-3 | 0.188 | -20.36944 | 0.426 | 20.00000 | 0.545 | -10.00000 | 0.670 | -8.33333 | 0.733 | -5.35804 | 0.642 | -7.40833 |
| Insomnia | 1-3 | 0.375 | -12.96444 | 0.455 | 16.66800 | 0.112 | -23.33400 | 0.538 | 11.11000 | 0.743 | -4.76143 | 0.819 | 2.38071 |
| Appetite loss | 1-2 | 0.801 | 3.03000 | 0.441 | -9.84448 | 0.333 | 11.90554 | 0.253 | -16.07682 | 0.800 | 3.02818 | 0.899 | 1.53679 |
| Appetite loss | 2-3 | 0.435 | -7.40778 | 0.374 | 13.33400 | 0.500 | -6.66700 | 0.635 | -5.55583 | 0.356 | -9.52429 | 0.363 | -11.11167 |
| Appetite loss | 1-3 | 1.000 | -0.00111 | 0.154 | 19.99700 | 1.000 | -0.00100 | 0.087 | -27.77583 | 1.000 | -0.00143 | 0.893 | 1.32079 |
| Constipation | 1-2 | 0.254 | 12.12182 | 0.186 | -19.04857 | 0.791 | 2.97929 | 0.192 | 16.47012 | 0.573 | 6.06000 | 0.138 | 15.89633 |
| Constipation | 2-3 | 0.171 | -9.25833 | 0.154 | 9.99900 | 0.154 | 9.99900 | 1.000 | 0.00000 | 1.000 | 0.00000 | 1.000 | 0.00000 |
| Constipation | 1-3 | 0.369 | 11.11222 | 1.000 | -0.00100 | 0.279 | 10.00100 | 0.131 | 22.22167 | 0.289 | 14.28714 | 0.111 | 14.81556 |
| Diarrhea | 1-2 | 0.748 | 3.02909 | 0.508 | -6.66667 | 0.542 | 5.95304 | 0.600 | -5.88235 | 0.096 | -15.15091 | 0.264 | -10.00000 |
| Diarrhea | 2-3 | 0.613 | -5.55611 | 0.099 | 26.66600 | 0.558 | 6.66500 | 0.404 | -11.11083 | 0.374 | -10.11946 | 0.320 | -12.96333 |
| Diarrhea | 1-3 | 1.000 | -0.00111 | 0.142 | 33.33200 | 0.848 | 3.33200 | 0.158 | -27.77583 | 0.357 | -14.88089 | 0.244 | -12.43373 |
| Financial difficulties | 1-2 | 0.637 | 6.06091 | 0.118 | -20.95305 | 0.322 | 13.09518 | 0.573 | -8.62718 | 0.637 | -6.06091 | 0.955 | -0.76964 |
| Financial difficulties | 1-3 | 0.318 | 16.66778 | 0.171 | -23.33400 | 0.705 | 6.66600 | 0.688 | -8.33250 | 0.974 | 0.59518 | 0.925 | -1.05810 |
| Swallow | 1-2 | 0.075 | 18.74800 | 0.302 | -12.38076 | **0.017*** | 22.15460 | 0.158 | -18.62494 | 0.603 | 5.68235 | 0.866 | 1.86031 |
| Swallow | 2-3 | 0.402 | -11.57389 | 0.595 | 11.66700 | 0.265 | -15.83400 | 0.685 | -6.94500 | 0.586 | -7.44054 | 0.603 | -9.25944 |
| Swallow | 1-3 | 0.584 | 7.86944 | 0.739 | 5.00100 | 0.502 | 9.99900 | 0.120 | -20.18300 | 0.125 | 16.47614 | 0.815 | -2.64563 |
| Sense | 1-2 | 0.330 | 12.12091 | 0.128 | -20.00048 | 0.716 | 4.76018 | 0.232 | -17.64600 | 0.408 | 9.97619 | 0.523 | 7.95082 |
| Sense | 2-3 | 0.232 | -14.81556 | 0.448 | 14.99900 | 0.446 | -10.00000 | 0.790 | -4.16583 | 0.346 | -11.60661 | 0.299 | -12.96222 |
| Sense | 1-3 | 0.897 | 1.85056 | 0.571 | -8.33400 | 0.910 | 1.66500 | **0.030*** | -24.25900 | 0.242 | 11.23962 | 0.252 | -10.18357 |
| Speech | 1-2 | 0.116 | 17.17091 | 0.707 | -4.54990 | 0.481 | 6.35071 | 0.475 | 9.54329 | 0.643 | -5.47045 | 0.564 | 6.83850 |
| Speech | 2-3 | 0.225 | -4.93778 | 0.242 | 8.88800 | 0.829 | -1.11100 | 0.538 | -3.70333 | 0.483 | -3.37268 | 0.479 | -4.32056 |
| Speech | 1-3 | 0.349 | 12.96222 | 0.820 | 3.33300 | 0.490 | 10.00200 | 0.872 | 2.78000 | 0.604 | 5.64076 | 0.640 | -5.20151 |
| Social eating | 1-2 | 0.303 | 10.60545 | 0.209 | -13.81048 | 0.375 | 9.52429 | 0.716 | -7.25365 | 0.249 | -11.99323 | 0.349 | -9.16482 |
| Social eating | 2-3 | 0.301 | -10.18611 | 0.529 | 10.00000 | 0.329 | -10.00100 | 0.735 | -4.16667 | 0.510 | -7.14286 | 0.518 | -8.33333 |
| Social eating | 1-3 | 0.847 | -2.78111 | 0.824 | -3.33500 | 0.781 | 4.16800 | 0.073 | -29.86083 | 0.819 | -2.27129 | 0.082 | -16.99698 |
| Social contact | 1-2 | 0.412 | 9.69818 | 0.401 | -10.66419 | 0.322 | 12.14214 | 0.956 | -0.78706 | 0.977 | -0.35015 | 0.442 | 9.33600 |
| Social contact | 2-3 | 0.435 | -2.22222 | 0.374 | 4.00000 | 0.500 | -2.00000 | 0.635 | -1.66667 | 0.356 | -2.85714 | 0.363 | -3.33333 |
| Social contact | 1-3 | 0.375 | 11.11333 | 0.649 | -5.99900 | 0.444 | 10.00000 | 0.591 | -8.33583 | 0.200 | 11.21379 | 0.526 | 5.76881 |

**Table S1.2**: Factors effecting change of QoL and symptom burdens (n=15)

|  |  | Age | | Gender | | Operation | | RT dose | | RT method | | Swallowing rehabilitation | |
| --- | --- | --- | --- | --- | --- | --- | --- | --- | --- | --- | --- | --- | --- |
|  | Time interval | p | Mean Difference | p | Mean Difference | p | Mean Difference | p | Mean Difference | p | Mean Difference | p | Mean Difference |
| Global | 1-2 | 0.531 | 7.22333 | 0.118 | -18.00200 | 0.504 | -8.00000 | 0.254 | 15.83417 | 0.748 | -3.66018 | 0.672 | -4.90667 |
| Global | 2-3 | 0.185 | -6.94500 | 0.437 | -3.33300 | 0.437 | -3.33300 | 0.494 | 3.47250 | 0.716 | 1.48768 | 0.577 | 2.31444 |
| Global | 1-3 | 0.983 | 0.27833 | 0.095 | -21.33500 | 0.395 | -11.33300 | 0.211 | 19.30667 | 0.865 | -2.17250 | 0.842 | -2.59222 |
| Utility | 1-2 | 0.548 | -0.10079 | 0.810 | 0.04264 | 0.701 | 0.06492 | 0.099 | -0.46535 | 0.615 | -0.08847 | 0.615 | -0.08847 |
| Utility | 2-3 | 0.455 | -0.040079 | 0.229 | 0.065972 | 0.753 | 0.017063 | 0.768 | -0.028535 | 0.229 | -0.065972 | 0.229 | -0.065972 |
| Utility | 1-3 | 0.494 | -0.14087 | 0.616 | 0.10861 | 0.693 | 0.08198 | 0.163 | -0.49389 | 0.473 | -0.15444 | 0.473 | -0.15444 |
| Physical | 1-2 | 0.561 | 5.92611 | 0.192 | 11.33400 | 0.601 | -4.66800 | 0.213 | 12.78000 | 0.563 | -4.88250 | 0.546 | 5.18500 |
| Physical | 2-3 | 0.684 | -1.11111 | 0.114 | -5.99900 | 0.478 | -2.00000 | 0.053 | 3.33333 | 0.448 | 2.14107 | 0.357 | 2.96111 |
| Physical | 1-3 | 0.677 | 4.81500 | 0.675 | 5.33500 | 0.504 | -6.66800 | 0.157 | 16.11333 | 0.773 | -2.74143 | 0.393 | 8.14611 |
| Role | 1-2 | 0.779 | 3.70389 | 0.264 | 14.99900 | 0.604 | -4.99900 | 0.127 | 23.60917 | 0.580 | -7.14143 | 0.944 | 0.92667 |
| Role | 2-3 | 0.363 | -5.55500 | 0.374 | -6.66600 | 0.374 | -6.66600 | 0.635 | 2.77750 | 0.356 | 4.76143 | 0.363 | 5.55500 |
| Role | 1-3 | 0.897 | -1.85111 | 0.571 | 8.33300 | 0.425 | -11.66500 | 0.112 | 26.38667 | 0.865 | -2.38000 | 0.648 | 6.48167 |
| Emotional | 1-2 | 0.613 | -6.01889 | 0.376 | 10.83300 | 0.164 | -16.66800 | 0.850 | -2.77667 | 0.435 | -9.97071 | 0.406 | -12.50056 |
| Emotional | 2-3 | 0.435 | 0.92556 | 0.374 | -1.66600 | 0.500 | 0.83300 | 0.635 | 0.69417 | 0.356 | 1.19000 | 0.363 | 1.38833 |
| Emotional | 1-3 | 0.640 | -5.09333 | 0.414 | 9.16700 | 0.147 | -15.83500 | 0.877 | -2.08250 | 0.451 | -8.78071 | 0.418 | -11.11222 |
| Cognitive | 1-2 | 0.136 | -18.51833 | 0.202 | 16.66800 | 0.157 | -18.33300 | 0.174 | 20.83417 | 0.907 | 1.48446 | 0.721 | 4.62667 |
| Cognitive | 2-3 | 0.169 | 7.40778 | 0.178 | -13.33400 | 0.622 | -3.33200 | 0.484 | 5.55583 | 0.926 | 0.59661 | 0.777 | 1.85333 |
| Cognitive | 1-3 | 0.319 | -11.11056 | 0.777 | 3.33400 | **0.048** | -21.66500 | **0.039*** | 26.39000 | 0.852 | 2.08107 | 0.566 | 6.48000 |
| Social | 1-2 | 1.000 | -0.00056 | 0.489 | 10.00000 | 0.731 | -5.00000 | 0.413 | 13.88917 | 0.180 | -17.85607 | 0.313 | -13.88833 |
| Social | 2-3 | 0.435 | 3.70333 | 0.374 | -6.66600 | 0.500 | 3.33300 | 0.635 | 2.77750 | 0.356 | 4.76143 | 0.363 | 5.55500 |
| Social | 1-3 | 0.737 | 3.70278 | 0.771 | 3.33400 | 0.884 | -1.66700 | 0.203 | 16.66667 | 0.212 | -13.09464 | 0.445 | -8.33333 |
| Fatigue | 1-2 | 0.184 | 13.57833 | 0.413 | -8.89200 | 0.919 | 1.11300 | 0.775 | -3.70583 | 0.302 | 10.51768 | 0.283 | 11.11056 |
| Fatigue | 2-3 | 0.359 | -11.72833 | 0.348 | 18.88900 | 0.562 | -7.77800 | 0.683 | -6.48167 | 0.311 | -14.08732 | 0.329 | -16.04944 |
| Fatigue | 1-3 | 0.847 | 1.85000 | 0.306 | 9.99700 | 0.500 | -6.66500 | 0.379 | -10.18750 | 0.704 | -3.56964 | 0.605 | -4.93889 |
| Nausea & vomiting | 1-2 | 0.634 | 7.40667 | 0.758 | 4.99900 | 0.347 | 15.00100 | 0.510 | 12.49667 | 0.172 | 21.43000 | 0.082 | 29.62944 |
| Nausea & vomiting | 2-3 | 0.363 | -5.55500 | 0.374 | -6.66600 | 0.165 | -6.66600 | 0.635 | 2.77750 | 0.356 | 4.76143 | 0.363 | 5.55500 |
| Nausea & vomiting | 1-3 | 0.907 | 1.85167 | 0.920 | -1.66700 | 0.612 | 8.33500 | 0.427 | 15.27417 | 0.074 | 26.19143 | **0.013*** | 35.18444 |
| Pain | 1-2 | 0.160 | 20.37000 | 0.486 | -10.83200 | 0.277 | 16.66900 | 0.282 | -19.44333 | 0.111 | 22.47036 | 0.141 | 21.29667 |
| Pain | 2-3 | 0.363 | -1.39000 | 0.374 | -1.66800 | 0.374 | -1.66800 | 0.635 | 0.69500 | 0.356 | 1.19143 | 0.363 | 1.39000 |
| Pain | 1-3 | 0.188 | 18.98000 | 0.414 | -12.50000 | 0.324 | 15.00100 | 0.294 | -18.74833 | 0.087 | 23.66179 | 0.110 | 22.68667 |
| Dyspnea | 1-2 | 0.315 | -16.66667 | 1.000 | 0.00100 | 1.000 | 0.00100 | 0.339 | 8.33333 | 0.302 | 14.28571 | 0.315 | 16.66667 |
| Dyspnea | 2-3 | 0.363 | -5.55500 | 0.500 | 3.33300 | 0.500 | 3.33300 | 0.635 | 2.77750 | 0.351 | -4.16625 | 0.435 | -3.70333 |
| Dyspnea | 1-3 | 0.070 | -22.22167 | 0.806 | 3.33400 | 0.806 | 3.33400 | 0.166 | 11.11083 | 0.460 | 10.11946 | 0.415 | 12.96333 |
| Insomnia | 1-2 | 0.435 | 7.40500 | 0.808 | -3.33200 | 0.165 | -13.33400 | 0.079 | 19.44333 | 0.950 | 0.59661 | 0.234 | 11.11167 |
| Insomnia | 2-3 | 0.188 | -20.36944 | 0.426 | 20.00000 | 0.545 | -10.00000 | 0.670 | -8.33333 | 0.733 | -5.35804 | 0.642 | -7.40833 |
| Insomnia | 1-3 | 0.375 | -12.96444 | 0.455 | 16.66800 | 0.112 | -23.33400 | 0.538 | 11.11000 | 0.743 | -4.76143 | 0.803 | 3.70333 |
| Appetite loss | 1-2 | 0.590 | 7.40667 | 0.641 | 6.66300 | 0.641 | 6.66600 | 0.173 | -22.22000 | 0.478 | 9.52286 | 0.415 | 11.11000 |
| Appetite loss | 2-3 | 0.435 | -7.40778 | 0.374 | 13.33400 | 0.500 | -6.66700 | 0.635 | -5.55583 | 0.356 | -9.52429 | 0.363 | -11.11167 |
| Appetite loss | 1-3 | 1.000 | -0.00111 | 0.154 | 19.99700 | 1.000 | -0.00100 | 0.087 | -27.77583 | 1.000 | -0.00143 | 1.000 | -0.00167 |
| Constipation | 1-2 | 0.140 | 20.37056 | 0.500 | -10.00000 | 1.000 | 0.00200 | 0.192 | 22.22167 | 0.302 | 14.28714 | 0.053 | 25.92667 |
| Constipation | 2-3 | 0.171 | -9.25833 | 0.154 | 9.99900 | 0.154 | 9.99900 | 1.000 | 0.00000 | 1.000 | 0.00000 | 1.000 | 0.00000 |
| Constipation | 1-3 | 0.369 | 11.11222 | 1.000 | -0.00100 | 0.279 | 10.00100 | 0.131 | 22.22167 | 0.289 | 14.28714 | 0.068 | 25.92667 |
| Diarrhea | 1-2 | 0.510 | 5.55500 | 0.446 | 6.66600 | 0.705 | -3.33300 | 0.091 | -16.66500 | 0.566 | -4.76143 | 0.510 | -5.55500 |
| Diarrhea | 2-3 | 0.613 | -5.55611 | 0.099 | 26.66600 | 0.558 | 6.66500 | 0.404 | -11.11083 | 0.374 | -10.11946 | 0.320 | -12.96333 |
| Diarrhea | 1-3 | 1.000 | -0.00111 | **0.037*** | 33.33200 | 0.848 | 3.33200 | 0.158 | -27.77583 | 0.357 | -14.88089 | 0.256 | -18.51833 |
| Financial difficulties | 1-2 | 0.318 | 16.66778 | 0.171 | -23.33400 | 0.705 | 6.66600 | 0.688 | -8.33250 | 0.972 | 0.59518 | 0.913 | 1.85167 |
| Financial difficulties | 1-3 | 0.318 | 16.66778 | 0.171 | -23.33400 | 0.705 | 6.66600 | 0.688 | -8.33250 | 0.972 | 0.59518 | 0.913 | 1.85167 |
| Swallow | 1-2 | 0.190 | 19.44333 | 0.675 | -6.66600 | 0.086 | 25.83300 | **0.029*** | -37.49667 | 0.215 | 19.04679 | 0.484 | 10.64833 |
| Swallow | 2-3 | 0.402 | -11.57389 | 0.595 | 11.66700 | 0.265 | -15.83400 | 0.685 | -6.94500 | 0.586 | -7.44054 | 0.603 | -9.25944 |
| Swallow | 1-3 | 0.584 | 7.86944 | 0.739 | 5.00100 | 0.502 | 9.99900 | **0.003*** | -44.44167 | 0.407 | 11.60625 | 0.923 | 1.38889 |
| Sense | 1-2 | 0.374 | 16.66611 | 0.225 | -23.33300 | 0.553 | 11.66500 | 0.103 | -36.11083 | 0.314 | 18.45464 | 0.733 | 6.48389 |
| Sense | 2-3 | 0.232 | -14.81556 | 0.448 | 14.99900 | 0.446 | -10.00000 | 0.790 | -4.16583 | 0.346 | -11.60661 | 0.420 | -12.96222 |
| Sense | 1-3 | 0.897 | 1.85056 | 0.571 | -8.33400 | 0.910 | 1.66500 | 0.008 | -40.27667 | 0.623 | 6.84804 | 0.648 | -6.47833 |
| Speech | 1-2 | 0.241 | 17.90000 | 0.733 | -5.55500 | 0.339 | 11.11300 | 0.735 | 6.48333 | 0.887 | 2.18393 | 0.813 | 3.70556 |
| Speech | 2-3 | 0.225 | -4.93778 | 0.242 | 8.88800 | 0.829 | -1.11100 | 0.538 | -3.70333 | 0.483 | -3.37268 | 0.479 | -4.32056 |
| Speech | 1-3 | 0.349 | 12.96222 | 0.820 | 3.33300 | 0.490 | 10.00200 | 0.872 | 2.78000 | 0.932 | -1.18875 | 0.965 | -0.61500 |
| Social eating | 1-2 | 0.577 | 7.40500 | 0.328 | -13.33500 | 0.297 | 14.16900 | 0.097 | -25.69417 | 0.768 | -3.86679 | 0.138 | -18.98000 |
| Social eating | 2-3 | 0.301 | -10.18611 | 0.529 | 10.00000 | 0.329 | -10.00100 | 0.735 | -4.16667 | 0.510 | -7.14286 | 0.518 | -8.33333 |
| Social eating | 1-3 | 0.847 | -2.78111 | 0.824 | -3.33500 | 0.781 | 4.16800 | 0.073 | -29.86083 | 0.433 | -11.00964 | **0.040*** | -27.31333 |
| Social contact | 1-2 | 0.358 | 13.33556 | 0.510 | -9.99900 | 0.428 | 12.00000 | 0.711 | -6.66917 | 0.278 | 15.36000 | 0.318 | 14.44778 |
| Social contact | 2-3 | 0.435 | -2.22222 | 0.374 | 4.00000 | 0.500 | -2.00000 | 0.635 | -1.66667 | 0.356 | -2.85714 | 0.363 | -3.33333 |
| Social contact | 1-3 | 0.375 | 11.11333 | 0.649 | -5.99900 | 0.444 | 10.00000 | 0.591 | -8.33583 | 0.307 | 12.50286 | 0.375 | 11.11444 |
