# Supplementary material for: Improved quality of life in head and neck cancer patients treated with modern arc radiotherapy techniques – A prospective longitudinal analysis
Source: Front Oncol. 2024 Sep 23;14:1424034. doi: 10.3389/fonc.2024.1424034 (PMC11456567; doi:10.3389/fonc.2024.1424034)
Supplement: Supplementary file 2 [file Table2.docx]

**Table S2.1**: Characteristics of patients with data at all three time points

| **Participants (n=15)** | **No. (%)** |
| --- | --- |
| ***Age, median (range), years*** | 56 (27-80) |
| ***Gender*** |  |
| Male | 10 (66.7) |
| Female | 5 (33.3) |
| ***Primary site*** |  |
| Oral cavity | 5 (33.3) |
| Nasopharynx | 2 (13.3) |
| Oropharynx | 2 (13.3) |
| Larynx/ Hypopharynx | 4 (26.7) |
| Other | 2 (13.3) |
| ***Cancer stage (AJCC 8^th^)*** |  |
| I-II | 8 (53.3) |
| III-IV | 7 (46.7) |
| ***Treatment category*** |  |
| Surgery plus adjuvant | 10 (66.7) |
| CCRT | 7 (46.7) |
| RT only | 3 (20.0) |
| Nonsurgical | 5 (33.3) |
| CCRT | 3 (20.0) |
| RT only | 2 (13.3) |
| ***RT dose*** |  |
| 6600 ~ 7000 cGy | 12 (80.0) |
| 6000 ~ 6400 cGy | 3 (20.0) |
| ***Techniques applied for radiotherapy*** |  |
| VMAT | 8 (53.3) |
| HT | 7 (46.7) |
| ***Swallow rehabilitation*** | 6 (40.0) |
| ***Patient services*** | 12 (80.0) |

Abbreviations: CCRT: concurrent chemoradiation therapy; HT: helical tomotherapy; RT: radiotherapy; VMAT: volumetric modulated arc therapy

**Table S2.2**: Comparison of global QOL, utility using multiple variables in patients with data at all three time points (n=15)

|  | ***Global QOL*** | |  | ***Utility*** | |
| --- | --- | --- | --- | --- | --- |
|  | Mean (SD) | P value |  | Mean (SD) | P value |
| ***Age*** |  |  |  |  |  |
| ≤ 55 y/o | 47.69 (24.39) | .300 |  | 0.31 (0.39) | .757 |
| > 55 y/o | 66.48 (30.02) |  |  | 0.51 (0.30) |  |
| ***Gender*** |  |  |  |  |  |
| Male | 56.11 (27.76) | .135 |  | 0.49 (0.38) | .257 |
| Female | 64.67 (31.94) |  |  | 0.32 (0.25) |  |
| ***Tumor site*** |  |  |  |  |  |
| Oral cavity | 47.78 (25.09) | .163 |  | 0.57 (0.36) | .606 |
| Nasopharynx | 62.50 (30.62) |  |  | 0.50 (0.07) |  |
| Oropharynx | 33.33 (18.26) |  |  | 0.13 (0.25) |  |
| Larynx/ Hypopharynx | 83.61 (23.15) |  |  | 0.44 (0.38) |  |
| Other | 59.72 (24.95) |  |  | 0.18 (0.32) |  |
| ***Cancer stage*** |  |  |  |  |  |
| I-II | 66.11 (29.20) | .123 |  | 0.40 (0.32) | .882 |
| III-IV | 50.79 (27.50) |  |  | 0.47 (0.39) |  |
| ***Chemotherapy*** |  |  |  |  |  |
| Yes | 47.22 (25.83) | **.010*** |  | 0.49 (0.36) | .336 |
| No | 82.44 (20.00) |  |  | 0.31 (0.31) |  |
| ***Operation*** |  |  |  |  |  |
| Yes | 52.22 (27.85) | .119 |  | 0.40 (0.37) | .970 |
| No | 72.44 (27.75) |  |  | 0.48 (0.32) |  |
| ***RT dose*** |  |  |  |  |  |
| 6600 ~ 7000 cGy | 57.73 (30.41) | .561 |  | 0.40 (0.33) | .149 |
| 6000 ~ 6400 cGy | 63.89 (24.30) |  |  | 0.57 (0.45) |  |
| ***RT method*** |  |  |  |  |  |
| VMAT | 70.63 (27.15) | .141 |  | 0.38 (0.32) | .912 |
| HT | 45.63 (25.90) |  |  | 0.50 (0.39) |  |
| ***Swallowing rehabilitation*** |  |  |  |  |  |
| Yes | 40.74 (24.23) | .069 |  | 0.57 (0.36) | .760 |
| No | 71.11 (25.88) |  |  | 0.35 (0.32) |  |
| ***Time*** |  |  |  |  |  |
| 2 weeks post-RT | 40.56 (30.19) | **.001*** |  | 0.45 (0.36) | .734 |
| 3 months post-RT | 69.56 (23.19) |  |  | 0.41 (0.34) |  |
| 6 months post-RT | 66.78 (25.90) |  |  | 0.43 (0.37) |  |

**Table S2.3:** Change of QOL, utility and symptom burdens between different time points in patients with data at all three time points (n=15)

| *Paired t-test* | **2 weeks - 3 months** | | | |  | **2 weeks - 6 months** | | |  | **3months - 6 months** | | |
| --- | --- | --- | --- | --- | --- | --- | --- | --- | --- | --- | --- | --- |
|  | n | | Mean difference | P value |  | n | Mean difference | P value |  | n | Mean difference | P value |
| Global QOL | 15 | -29.000 | | **<.001*** |  | 15 | -26.222 | **<.001*** |  | 15 | 2.778 | .173 |
| Utility | 14 | 0.053 | | .506 |  | 14 | 0.027 | .781 |  | 14 | -0.022 | .314 |
| Physical | 15 | -9.778 | | **.028*** |  | 15 | -7.111 | .135 |  | 15 | 2.667 | .054 |
| Role | 15 | -3.333 | | .595 |  | 15 | -1.111 | .869 |  | 15 | 2.222 | .334 |
| Emotional | 15 | -7.778 | | .182 |  | 15 | -7.222 | .176 |  | 15 | 0.556 | .334 |
| Cognitive | 15 | -5.556 | | .371 |  | 15 | -1.111 | .836 |  | 15 | 4.444 | .164 |
| Social | 15 | -16.667 | | **.023*** |  | 15 | -14.444 | **.013*** |  | 15 | 2.222 | .334 |
| Fatigue | 15 | 11.852 | | **.030*** |  | 15 | 6.667 | .156 |  | 15 | -5.185 | .404 |
| Nausea & vomiting | 15 | 10.000 | | .189 |  | 15 | 12.222 | .119 |  | 15 | 2.222 | .334 |
| Pain | 15 | 20.000 | | **.023*** |  | 15 | 16.667 | **.013*** |  | 15 | -3.333 | .334 |
| Dyspnea | 15 | 6.667 | | .334 |  | 15 | 8.889 | .164 |  | 15 | 2.222 | .334 |
| Insomnia | 15 | 4.444 | | .334 |  | 15 | -2.222 | .751 |  | 15 | -6.667 | .384 |
| Appetite loss | 15 | 4.444 | | .499 |  | 15 | 0.000 | 1 |  | 15 | -4.444 | .334 |
| Constipation | 15 | 6.667 | | .334 |  | 15 | 6.667 | .271 |  | 15 | 0 | 1 |
| Diarrhea | 15 | -2.222 | | .582 |  | 15 | -11.111 | .173 |  | 15 | -8.889 | .104 |
| Financial difficulties | 15 | 4.444 | | .582 |  | 15 | 4.444 | .582 |  | 15 | 0 | 1 |
| Swallow | 15 | 25.556 | | **.003*** |  | 15 | 20.000 | **.010*** |  | 15 | -5.556 | .406 |
| Sense | 15 | 4.444 | | .623 |  | 15 | 1.111 | .869 |  | 15 | -3.333 | .582 |
| Speech | 15 | 16.296 | | **.042*** |  | 15 | 13.333 | .060 |  | 15 | -2.963 | .217 |
| Social eating | 15 | 12.778 | | .058 |  | 15 | 9.444 | .180 |  | 15 | -3.333 | .486 |
| Social contact | 15 | 14.667 | | .050 |  | 15 | 13.333 | **.040*** |  | 15 | -1.333 | .334 |
| Sexuality | 15 | 10.000 | | .308 |  | 15 | 0.000 | 1 |  | 15 | -10.000 | .167 |
| Teeth | 15 | 13.333 | | .138 |  | 15 | 0.000 | 1 |  | 15 | -13.333 | .111 |
| Open mouth | 15 | 8.889 | | .262 |  | 15 | 4.444 | .582 |  | 15 | -4.444 | .334 |
| Dry mouth | 15 | 11.111 | | .136 |  | 15 | 4.444 | .582 |  | 15 | -6.667 | .189 |
| Saliva | 15 | 28.889 | | **.001*** |  | 15 | 22.222 | **.003*** |  | 15 | -6.667 | .384 |
| Cough | 15 | 15.556 | | **.014*** |  | 15 | 11.111 | .265 |  | 15 | -4.444 | .546 |
| Ill | 15 | 17.778 | | **.041*** |  | 15 | 17.778 | **.015*** |  | 15 | 0 | 1 |
| Painkiller | 15 | 33.333 | | .055 |  | 15 | 33.333 | **.019*** |  | 15 | 0 | 1 |
| Nutrition | 15 | 13.333 | | .433 |  | 15 | 33.333 | **.019*** |  | 15 | 20.000 | .082 |
| NG | 15 | 0.000 | | 1 |  | 15 | 0.000 | 1 |  | 15 | 0 | 1 |
| Weight loss | 15 | 46.667 | | **.004*** |  | 15 | 33.333 | .055 |  | 15 | -13.333 | .164 |
| Weight gain | 15 | -13.333 | | .334 |  | 15 | -13.333 | .334 |  | 15 | 0 | 1 |
